# Brain Amide Proton Transfer Imaging of Rat With Alzheimer’s Disease Using Saturation With Frequency Alternating RF Irradiation Method

**DOI:** 10.3389/fnagi.2019.00217

**Published:** 2019-08-22

**Authors:** Runrun Wang, Peidong Chen, Zhiwei Shen, Guisen Lin, Gang Xiao, Zhuozhi Dai, Bingna Zhang, Yuanfeng Chen, Lihua Lai, Xiaodan Zong, Yan Li, Yanyan Tang, Renhua Wu

**Affiliations:** ^1^Department of Medical Imaging, The Second Affiliated Hospital, Shantou University Medical College, Shantou, China; ^2^Philips Healthcare, Shantou, China; ^3^Department of Mathematics and Statistics, Hanshan Normal University, Chaozhou, China; ^4^Translational Medicine, The Second Affiliated Hospital, Shantou University Medical College, Shantou, China

**Keywords:** Alzheimer’s disease, amide proton transfer, saturation with alternating frequency RF irradiation, chemical exchange saturation transfer, magnetic resonance imaging

## Abstract

Amyloid-β (Aβ) deposits and some proteins play essential roles in the pathogenesis of Alzheimer’s disease (AD). Amide proton transfer (APT) imaging, as an imaging modality to detect tissue protein, has shown promising features for the diagnosis of AD disease. In this study, we chose 10 AD model rats as the experimental group and 10 sham-operated rats as the control group. All the rats underwent a Y-maze test before APT image acquisition, using saturation with frequency alternating RF irradiation (APT_SAFARI_) method on a 7.0 T animal MRI scanner. Compared with the control group, APT (3.5 ppm) values of brain were significantly reduced in AD models (*p* < 0.002). The APT_SAFARI_ imaging is more significant than APT imaging (*p* < 0.0001). AD model mice showed spatial learning and memory loss in the Y-maze experiment. In addition, there was significant neuronal loss in the hippocampal CA1 region and cortex compared with sham-operated rats. In conclusion, we demonstrated that APT imaging could potentially provide molecular biomarkers for the non-invasive diagnosis of AD. APT_SAFARI_ MRI could be used as an effective tool to improve the accuracy of diagnosis of AD compared with conventional APT imaging.

## Introduction

Alzheimer’s disease (AD) is the most prevalent neurodegenerative disease in the world, which is characterized with progressive memory decline (Chen et al., 2018). Currently there is no definitive diagnosis or effective treatment for AD (Goozee et al., 2017). Many pathogenic mechanisms have been reported, including the accumulation of amyloid plaques, neuronal loss, neurofibrillary tangles (NFTs), excessive acetylcholinesterase activity and neurovascular dysfunction (Vanderstichele et al., 2006). Biomarkers based on protein aggregation play important roles in evaluating AD. The reliable AD model rat can be made by intracerebroventricular (icv) injection of well-characterized toxic soluble Aβ species into rat brain (Kasza et al., 2017). The injection of Aβ species in rat induced loss of learning and memory behavior, which could be detected using the Y-maze (Hwang et al., 2017). The rat model of AD was used to observe the effect of drug therapy (Fahanik-Babaei et al., 2018) and was often used to establish and validate biomarkers as a surrogate for patients (Prestia et al., 2018) as well.

Magnetic resonance imaging (MRI) is essential for early diagnosis of AD (Matsuda, 2017), including conventional MRI, diffusion tension imaging (DTI) (Promteangtrong et al., 2015), proton magnetic resonance spectroscopy (MRS) (Zhang N. et al., 2014). The accuracy of AD diagnosis may be increased using advanced MRI techniques. Up to now, further reliable imaging technique for early AD diagnosis is still desired. Amide proton transfer (APT) imaging based on chemical exchange saturation transfer (CEST) is a novel molecular MRI technique (Kamimura et al., 2018), by which low-concentration endogenous mobile proteins and peptides in tissue could be detected non-invasively (Xu et al., 2014). Furthermore, multiple sources of exchanging magnetization, like amide (Lin et al., 2018), amine (Zhang et al., 2018) and hydroxyl protons from macromolecules and various protons (Kanazawa et al., 2018), could also be detected. AD is associated with the accumulation of abnormal proteins in the central nervous system (Li et al., 2017). However, the quantitative method to detect protein *in vivo* is limited. To our knowledge, few studies have been reported to diagnose AD by APT method.

Zaiss and Bachert (2013) proved from algorithms and theoretical formulas that chemical exchange observed by NMR saturation transfer (CEST) and spin-lock (SL) experiments provided a MRI contrast by indirect detection of exchanging protons. A comprehensive signature of protein unfolding detectable by CEST was observed in a set of model solutions containing BSA and in yeast cells (Goerke et al., 2015). Zollner et al. (2018) demonstrated that APT-weighted CEST imaging is sensitive to ammonia introduced protein denaturation. Using APT to detect tau-pathology in regions of low NFT density is also a method to study AD in mouse model of tauopathy (rTg4510) (Wells et al., 2015). In Chen et al. (2019) study, a new sequence (radial-sampling steady-state sequence based ultrashort echo time readout) was used to image the contributions from mobile proteins at the frequency offsets for both aliphatic proton (–3.6 ppm) and protein amide proton (+3.6 ppm) signals. Their results showed significantly reduced ΔST (–3.6) signal in AD mouse, which was more sensitive. In this study, we use the AD model (based on intracerebrovascular injection of the beta amyloid 1-40). Target APT imaging could potentially provide molecular biomarkers for diagnosis of AD. These results suggested that APT_SAFARI_ MRI could be used as an effective tool to improve the accuracy for the diagnosis of AD.

In this study, we hypothesized that the accumulation of abnormal cytoplasmic proteins in some specific cerebral areas was associated with low APT signal. Meanwhile, saturation with frequency alternating radiofrequency irradiation (SAFARI) method was used to improve accuracy of APT signal by removing the direct water saturation (DS) effect, magnetization transfer (MT) effect and MT asymmetry (Scheidegger et al., 2011).

## Materials and Methods

### Alzheimer Disease Model Preparation

Sprague-Dawley (SD) male rats weighting 275 ± 25 g (aged 10–12 weeks) were purchased from the Animal Center of Shantou University Medical College (Guangdong, China). All animal experiments were performed according to the guidelines of the National Institutes of Health guide and approved by the Ethics Committee of Shantou University Medical College. The rats were randomly divided into two groups: sham-operated control group rats (*n* = 10) and AD model group rats (*n* = 10). Both groups were placed in a geomagnetic environment. The rats were housed in an air-conditioned room with a constant temperature (22 ± 1°C), humidity (50 ± 10%), and were kept under reversed light/dark (12 h each) cycle.

At Sigma-Aldrich (St. Louis, MO, United States), we purchased Aβ1–40. To obtain aggregated Aβ1-40, Aβ1-40 was dissolved at the concentration of 1 g/L in distilled water and was incubated for 48 h at 37°C. Then it was diluted to the final concentration with saline just before the experiments (Guerra de Souza et al., 2018). After aggregation, the sample was stored at 4°C. The SD rats received icv injection of Aβ1-40 as described before (Rasool et al., 2018). Briefly, the rats were anesthetized with 3 mg/ml sodium pentobarbital (1 ml/100 g, i.p. body weight). Then they were placed in stereotaxic apparatus. For a single icv injection of aggregated Aβ1-40, a 28-G needle (stainless-steel) was inserted into lateral ventricular (1.0 mm lateral, 3.6 mm central to bregma and 0.8 mm posterior). And then AD model group rats were administered with Aβ1-40 10 μg per rat (1 mg/ml) using Hamilton microsyringe at a speed of 0.6 μl/min. Sham-operated control group rats were given the same volume of normal saline. The cannula was left for 2–3 min after the injection to facilitate drug diffusion. The wound as an additional antiseptic measure was then sealed with sterile wax.

### Behavioral Testing

All rats underwent Y-maze testing 14 days after the model was built. The Y-maze test was used to assess the spatial learning and memory of the rats (Zhang L. et al., 2014). The spontaneous alternation behavior, the time spent in the new arm, total distance and the total new arm distance were measured to assess the learning ability of the rats (Conrad et al., 2003). Behavioral studies were carried out 2 h after last work between 9 am and 5 pm in a quiet room. Before testing the next rat, the device was cleaned with 10% ethanol. The tests were recorded using a video camera and later scored by a trained observer who was blind to the grouping of the rats. Each rat was placed at the start arm and moved freely through the maze for 10 min. An alternation was defined as successive entries into all three arms on consecutive choices (i.e., BCA, ABC, or CAB but not ABA). Spontaneous alternation, as a measure of cognitive functions, assesses short-term spatial memory. The percentage of spontaneous alternation was calculated as alternation rate (%) = 100 × [1 – mistake number/(total number – 2)] (Bak et al., 2017). The second test aimed to test spatial learning. The three arms were set as the starting arm (animal entry), the common arm and the new arm. In the first step, which was the acquisition period, the new arm was closed, and the rats were free to explore to the other two arms for 3 min. Two hours later, the second step (recall phase) began. All the arms were opened, the animals were free to move for 3 min in the three arms. The time and distance of exploration in each arm were recorded.

### MRI Experiments

After behavioral testing, all AD model rats were scanned 15 days after model was built. All images were acquired on a 7.0T horizontal bore small animal MR scanner (Agilent Technologies, Santa Clara, CA, United States) with a standard 9563 volume coil for transmission and reception. Parameters of T2WI MRI were as follows. TR = 3,140 ms, TE = 37 ms, FOV = 40 mm × 40 mm, matrix = 240 × 320, and slice thickness = 1 mm. We scanned 6 slices for T2w images and selected the largest slice of the hippocampus for shimming.

The main magnetic field (B_0_) was shimmed. The axial APT images were acquired using a single slice echo planar imaging (EPI) sequence with continuous wave (CW) pre-saturating RF irradiation. FOV = 35 × 35 mm, slice thickness = 3.5 mm, matrix size = 128 × 128, repetition time (TR) = 5,000 ms, echo time (TE) = 20 ms, and bandwidth = 267,000 Hz. The APT imaging and Z-spectra were acquired, which ranged from 5 to –5 ppm, with the use of a B1 of 1.3 μT (56 Hz) and a saturation time of 4 s. A saturation pulse was applied at 101 frequency offsets that cover the range of ±5 ppm and step of 0.1 ppm to contain around ±3.5 ppm of APT saturation peaks. The APT imaging was 8 min and 40 s. S_0_ was acquired at saturation frequency offset of 33.33 ppm as a reference image. Saturation with frequency alternating RF irradiation (SAFARI) was achieved by setting a dual frequency preparation of a gauss pulse saturation at ±3.5 ppm (Scheidegger et al., 2011). Then 101 frequency offsets images were detected using a gauss pulse saturation with the same range and step as CW pulse sequence. The total time of SAFARI imaging was 8 min and 45 s. The B_0_ and B1 fields were also measured, as well as T1 and T2 maps. T1 maps were acquired using the same geometry and spatial resolution as CEST MRI. An IR-FSEMS sequence with Inversion recovery time = 0.010, 0.023, 0.051, 0.115, 0.260, 0.588, 1.328, 3 s was used for T1 maps. While the T2 map was obtained by a multi-slice multi-echo (MSME) MRI with echo number = 16. Echo time = 8.2, 16.3, 24.5, 32.7, 40.8, 49.0, 57.2, 65.3, 73.5, 81.7, 89.8, 98.0, 106.2, 114.4, 122.5, 130.7 ms was used for T2 maps.

### Image Analysis

Images were analyzed in MATLAB (MathWorks, R2012b). For APT acquisition, we normalized voxels of images by the corresponding unsaturated reference image S_0_. Then B_0_ correction was performed for the z-spectrum scans according the water saturation shift referencing (WASSR) method (Kim et al., 2009). Evaluation of the APT effect by conventional MT ratio asymmetry analysis after B_0_ correction (Wada et al., 2016):

$${\text{MTR}}_{\text{asym}}=\frac{{\text{S}}_{\text{sat}}\,\left(-3.5\,\text{ppm}\right)-{\text{S}}_{\text{sat}}\,\left(+3.5\,\text{ppm}\right)}{{\text{S}}_{0}}$$

For the APT_SAFARI_ scans, calculated the quantitative maps of MTR_SAFARI_ as described previously (Scheidegger et al., 2011):

$${\text{MTR}}_{\text{SAFARI}}=\frac{{\text{S}}_{\text{sat}}\,\left(+3.5\,\text{ppm}\right)+{\text{S}}_{\text{sat}}\,\left(-3.5\,\text{ppm}\right)}{{\text{S}}_{0}}-\frac{{\text{S}}_{\text{sat}}\,\left(\text{SAFARI}\right)+{\text{S}}_{\text{sat}}\,\left({\text{SAFARI}}^{\prime }\right)}{{\text{S}}_{0}}$$

where S_*sat*_ (SAFARI) is the signal after alternating frequency irradiation and S_*sat*_ (SAFARI′) is a similar image but with the order of positive and negative frequencies reversed to minimize any system error related timing (Scheidegger et al., 2011).

The hippocampus is the primary structure affected in the early AD pathology which control the learning and cognitive function (Zhang et al., 2017). Therefore, we selected the largest slice of hippocampus on EPI-based image according to the high spatial resolution atlases exist for MRI (Johnson et al., 2010) as our APT slice. ROIs across all slices containing cortex, hippocampus and thalamus were manually drawn by the same expert with visual reference to a rat brain atlas. The T2w image demonstrates the ROIs in the coronal brain slices for the cortex (CX), hippocampus (HI), and thalamus (TH). The red dotted lines indicate the ROI tissues (Figure 3A). ROIs for the APT image were manually drawn on the EPI-based image (Figure 3B).

### Histology and Histomorphometry

After imaging scanning, rats were anesthetized and perfused *via* the left ventricle with 100 mL of 4% paraformaldehyde followed by 100 mL of normal saline at a flow rate of 3 mL/min. After perfusion, the brains were obtained and kept in 4% para formaldehyde (24 h) and embedded in paraffin before being dispatched for histology (Wang et al., 2018).

Axial slices (5 μm) were incubated at 55°C for 45 min. Hematoxylin-eosin (HE) staining was used to assesse neuropathology. Finally, the slices were examined under a Zeiss microscope (Zeiss Instruments Inc.).

The process of double-labeling immunofluorescence was as follows. Staining coverslips with 70 nm serial sections were done as previously described (Kay et al., 2013). Paraffin-embedded sections were rehydrated with reduced concentrations of ethanol and subjected to a standard antigen- retrieval procedure consisting of being microwaved in 5% goat serum for 20 min (ZLI-9056, China). The sections were cooled for about 40 min at 4°C. They were then blocked with 5% normal goat serum for 1 h at room temperature. Finally, they were incubated with the primary antibody overnight at 4°C. After 24 h, the sections were deparaffinized and washed through a series of xylene and ethanol to rehydrate. With anti-GFAP to label astrocytes, all primary antibodies were diluted in PBS. Slides were incubated for the secondary antibody and fluorescently labeled for 1 h. Finally, the coverslips were mounted on glass slides and then observed using the Zeiss laser confocal microscope.

### Statistical Analysis

All data were analyzed using the SPSS22.0. Imaging data of AD models and control subjects were compared using *t*-test for pairwise comparison. One-way analysis of variance (ANOVA) followed by multiple comparisons were used to investigate the associations between Y-maze. A level of *p* < 0.05 was considered as statistically significant for all tests.

## Results

### Y-Maze Test Results of AD Model Rat

**FIGURE 1:**
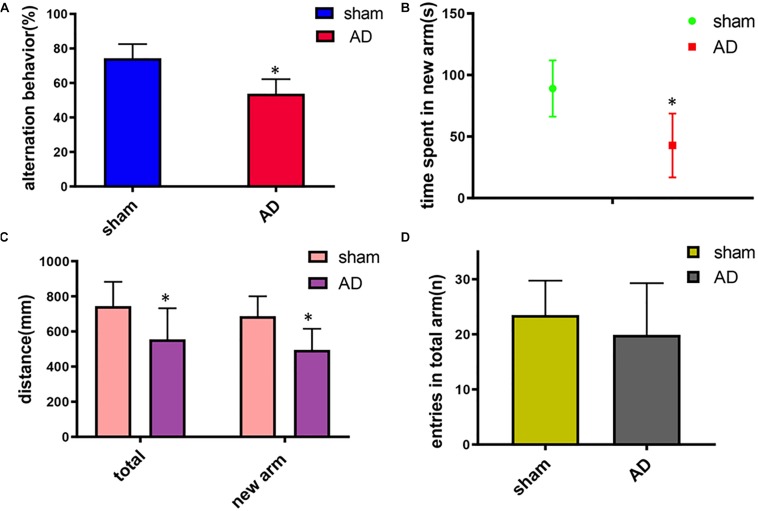
Learning and memory assessment of AD model group and sham operated control group measured by Y-maze. **(A)** Alternation behavior: AD model group had significantly decreased the spontaneous alternation behavior compared with the sham operated control group. **(B)** Time spent in the new arm: The time spent in the new arm of AD model group decreased significantly compared with the sham operated control group. **(C)** Total distance and the total new arm distance: AD model group had significant decrease of the total distance and the total new arm distance compared with the sham operated control group. **(D)** There was no significant difference between two groups on the numbers of arm entries. ^∗^*p* < 0.05.

In the Y-maze test, significant decrease of the spontaneous alternation was found in AD model group compared with the sham operated control group (*p* < 0.05, Figure 1A). Meanwhile, a significant decrease of the time, total distance and the total distance in the new arm were also found in AD model group compared with the sham-operated control group (*p* < 0.05, Figures 1B,C). There was no significant difference between the two groups in the numbers of arm entries (Figure 1D). These results demonstrated the AD model had loss spatial learning and memory.

### Results of APT Imaging and SAFARI Imaging

**FIGURE 2:**
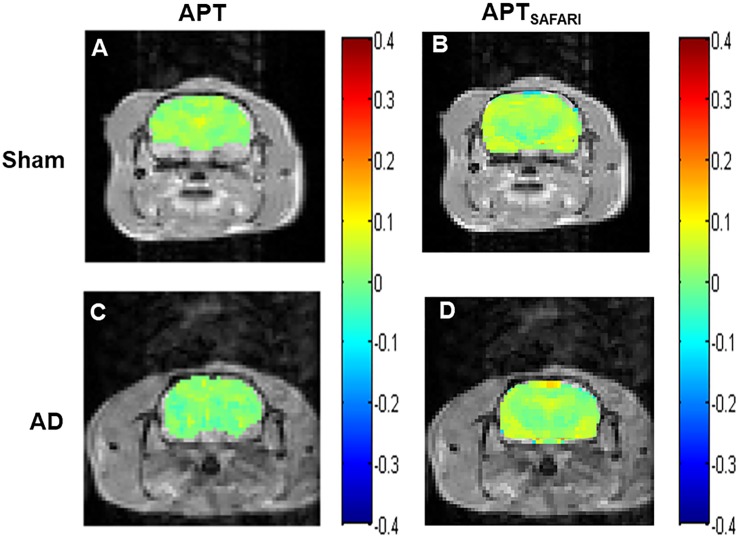
**(A)** APT imaging of sham operated control, **(B)** APT_*SAFARI*_ imaging of sham operated control, **(C)** APT imaging of AD model, **(D)** APT_*SAFARI*_ imaging of AD model.

**FIGURE 3:**
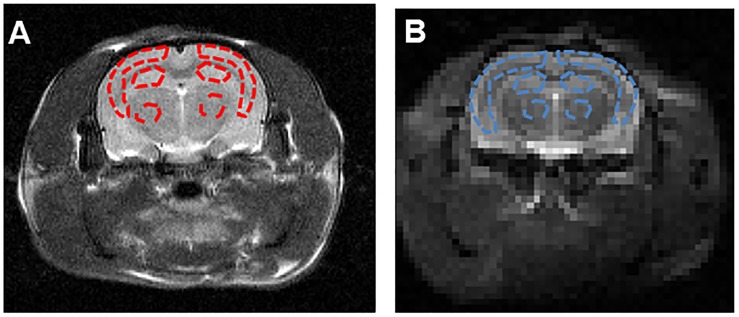
**(A)** The T2w image demonstrates the ROIs in the coronal brain slices for the cortex (CX), hippocampus (HI) and thalamus (TH). The red dotted lines indicate the ROI tissues. **(B)** ROIs for the APT image were manually drawn on the EPI-based image. The blue dotted lines indicate the ROI tissues.

**FIGURE 4:**
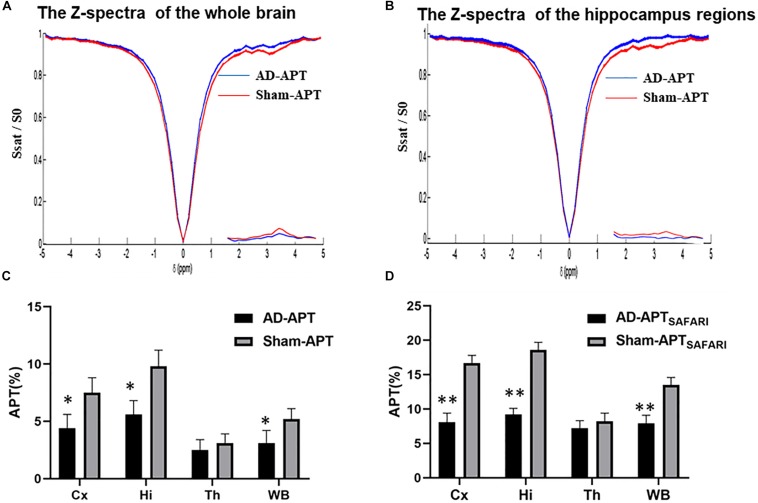
**(A)** The Z-spectra and MTR_*asym*_ curve between AD model and sham controls in the whole brain, **(B)** The Z-spectra and MTR_*asym*_ curve between AD model and sham controls in hippocampus regions, **(C)** The plot for the different region APT effect in AD model and sham groups, **(D)** The plot for the different region APT_*SAFARI*_ effect in AD model and sham groups. ^∗^*p* < 0.05, ^∗∗^*p* < 0.01.

The Z-spectra and MTR_*asym*_ curves showed that there were significant differences between AD models and sham operated controls in the whole brain (Figure 4A), there are more significant reductions when hippocampus regions are compared (Figure 4B). ΔMTR_*asym*_ was maximal with a 1.3 μT B_1_ power and the peak of MTR_*asym*_ curve was at 3.5 ppm. The APT maps of AD model and sham control were showed in the Figures 2A,C. The APT_*SAFARI*_ maps of AD model and sham control were showed in the Figures 2B,D. Image uniformity and image contrast of the SAFARI method were all better than that acquired from APT imaging. T2w anatomical only-image is added for the readers to understand the exact geometrical position of the selected slice (Figure 3A). ROIs for the APT image were manually drawn on the EPI-based image (Figure 3B). AD model rats (*n* = 10) had reduced APT effect compared to the sham groups. The APT effects of AD model rats were 4.7 ± 1.2, 5.9 ± 1.4, 2.78 ± 0.9, and 3.3 ± 1.1% at CX, HI, TH and whole brain (WB), respectively. APT effects at CX, HI and WB were lower than that in sham controls (7.5 ± 1.3%, 9.6 ± 1.5%, 5.2 ± 0.9%, *p* < 0.05). At TH, no significant differences in APT effect were observed between two groups (*p* > 0.05) (Figure 4C). The APT_*SAFARI*_ effects of AD model in above four regions were 8.5 ± 1.2, 9.6 ± 1.3, 7.7 ± 1.3, and 8.2 ± 1.2%, respectively. APT_*SAFARI*_ effects at CX, HI and WB, were lower than that in sham controls (16.8 ± 1.4%, 18.7 ± 1.1%, 13.5 ± 1.3%, *p* < 0.01). At TH, no significant differences in APT effect were observed at two groups (*p* > 0.05) (Figure 4D).

### Results of T1 and T2 Maps

**FIGURE 5:**
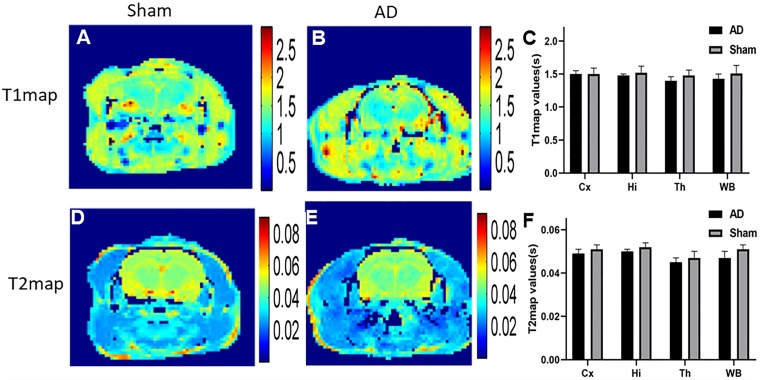
**(A)** T1 map of sham operated control, **(B)** T1 map of AD model, **(C)** The plot for the different region T1 map values in AD model and sham groups, **(D)** T2 map of sham operated control, **(E)** T2 map of AD model, **(F)** The plot for the different region T2 map values in AD model and sham groups.

In our study, in order to examine the possible difference of T1 and T2 maps between two groups (Figure 5), we scanned T1 and T2 maps. The T1 map values were found to be 1.43 ± 0.07 and 1.51 ± 0.12 s for the cortex of the AD rats and sham rats, respectively; while the T2 map values were 0.047 ± 0.003 s (AD rats) and 0.051 ± 0.002 s (sham rats). No significant difference was observed between AD and sham rats (*p* = 0.29 and 0.21 for the T1 and T2 map values, respectively). No significant differences in different regions of T1 and T2 map values were observed either between two groups (*p* > 0.05) (Figures 5C,F).

### Results of Histological Examinations

**FIGURE 6:**
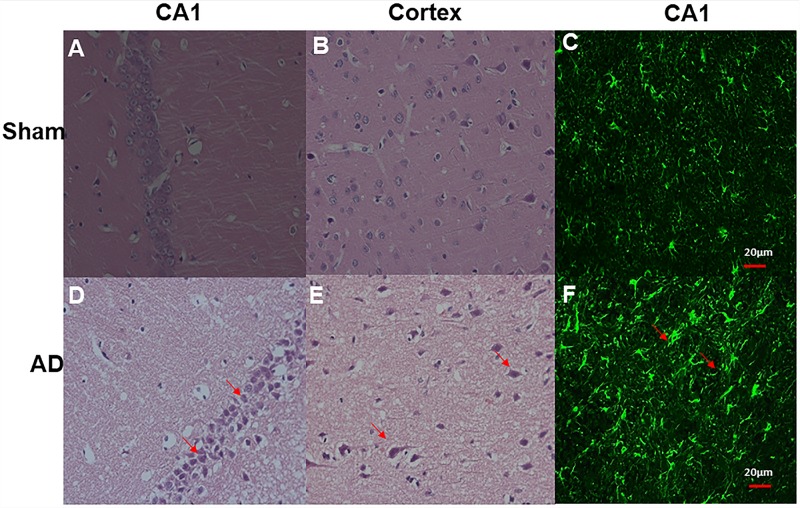
HE staining and double-labeling immunofluorescence and confocal microscopy of GFAP (green) of Hippocampal CA1 and cortex. HE staining **(A)** CA1 region of sham operated control, **(B)** Cortex of sham operated control, **(D)** CA1 region of AD model, **(E)** Cortex of AD model (above all original magnification × 40). AD model group showed significant neuronal loss in the hippocampus CA1 region and cortex (red arrows); Double-labeling immunofluorescence **(C)** Hippocampal CA1 region of sham operated control, **(F)** Hippocampal CA1 region of AD model; GFAP staining is strongly enhanced in reactive astrocytes identified by double-labeling immunofluorescence (red arrows) in AD model, Scale bars = 20 μm.

**FIGURE 7:**
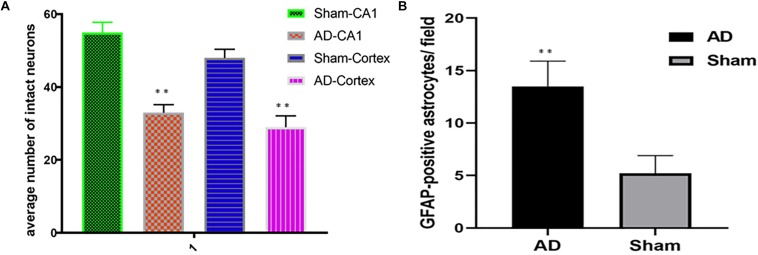
**(A)** HE staining, AD model group showed significant neuronal loss in the hippocampus CA1 region and cortex compared sham operated control, **(B)** Bar graphs of mean densities of GFAP-positive reactive astrocytes of AD model and sham group. ^∗∗^*p* < 0.01.

Significant changes in neuron morphology in each group were revealed in histological studies. For HE staining, the number of intact neurons in the hippocampal CA1 was markedly decreased in the AD model group compared with those in the sham operated control group (Figure 7A). The AD model group showed significant neuronal loss in the hippocampus CA1 region and cortex (Figures 6D,E, red arrows). GFAP staining was strongly enhanced in reactive astrocytes identified by double-labeling immunofluorescence in the AD model (Figure 6F, red arrows). Figures 6A–C are the pathological results of the corresponding region of sham operated controls. The total number of GFAP-positive astrocytes was expressed as the mean number per field of view. A significant increase in the number of GFAP-positive astrocytes was observed in the hippocampus CA1 region of the AD model, as compared to sham rats (Figure 7B).

### Correlation Analysis

**FIGURE 8:**
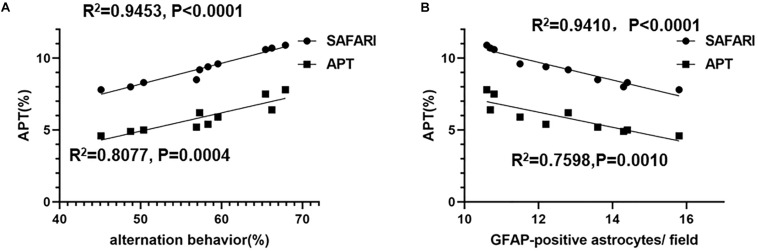
**(A)** The linear regression analysis of the correlation between alternation behavior (%) and the APT (%) of the hippocampus in AD model rats (*R*^2^ = 0.9453, *p* < 0.0001 and *R*^2^ = 0.8077, *p* = 0.0004 for the APT_*SAFARI*_ and APT values, respectively). **(B)** The linear correlation between the APT (%) and GFAP-positive astrocytes/field of the hippocampus in AD model rats (*R*^2^ = 0.9410, *p* < 0.0001 and *R*^2^ = 0.7598, *p* = 0.0010 for the APT_*SAFARI*_ and APT values, respectively).

The linear regression analysis revealed a positive correlation between alternation behavior (%) and the APT (%) of the hippocampus in AD model rats (*R*^2^ = 0.9453, *p* < 0.0001 and *R*^2^ = 0.8077, *p* = 0.0004 for the APT_*SAFARI*_ and APT values, respectively) (Figure 8A). A negative correlation between the APT (%) and GFAP-positive astrocytes/field of the hippocampus was found in AD model rats (*R*^2^ = 0.9410, *p* < 0.0001 and *R*^2^ = 0.7598, *p* = 0.0010 for the APT_*SAFARI*_ and APT values, respectively) (Figure 8B). In terms of goodness of fit, APT_*SAFARI*_ was better than APT for both behavior and pathology correlation. These results demonstrated that APT_*SAFARI*_ was a more sensitive method to detect CEST signal change compared with that using APT method.

## Discussion

In this study, we used both APT and APT_*SAFARI*_ (Scheidegger et al., 2011) methods to assess the APT value in AD models at 7.0T. These results indicate the APT is a potential method that can non-invasively visualize the protein concentration of AD *in vivo*. APT imaging is a novel molecular MRI technique for detecting endogenous mobile proteins. APT could be affected by many other factors, including tissue water content, pH, temperature, and the background MT effect (Togao et al., 2014). In our study, the results showed significantly reduced signal for the AD model compared to the control group, which is due to the effect of protein aggregation involved in AD (Chen et al., 2019). Several studies of APT have shown that the protein concentration is homogenous throughout the brain (Xu et al., 2016). APT is highly sensitive to changes of pH in tissue, although it is designed to provide a direct measurement of proton exchange. The pathology of AD also includes vascular compromise that can result in hypoperfusion, local tissue hypoxia, and acidosis (Eugenin et al., 2016). Brain acidification in AD patients has already been observed (Fang et al., 2010). This reduced pH results in a reduced rate of exchange of amide protons because the chemical exchange of the amide in the protein is base catalyzed (Zhang et al., 1995). Exchange of these amide protons with water results in a reduction in MR imaging signal that is highly pH-sensitive (Jin et al., 2017). In particular, it may serve as an important biomarker when evaluating the efficacy of novel therapeutics that target pH-sensitive pathways (Anand et al., 2014). Because of the reduced mobile protein content and decrease of pH, AD should have lower APT value than normal control. Our study also demonstrated this viewpoint.

The key to the SAFARI technology is the simultaneous application of RF radiation to acquire images at both the amide proton (ωs = +3.5 ppm) and control (–ωs) frequencies. There is a range of RF acquisition which the amide proton saturation is independent of power. SAFARI only needs to acquire three MR images, and the sum of these images can eliminate the symmetrical MT effects (Scheidegger et al., 2011). A series of SAFARI acquisitions may be used to more selectively detect specific endogenous biomolecules with a unique chemical exchange rate (Bateman et al., 2012). Compared with a conventional MTRasym measurement, the SAFARI method has the advantage of reducing the effect of MT, direct water saturation, and field inhomogeneity and measurement times.

T1 and T2 maps have emerged to be able to adequately identify the biochemical composition and changes of the cartilaginous tissue (Ying et al., 2019). These sequences also enable the direct quantification of T1, T2 values of the myocardium (Kim et al., 2017). In our study, no clear differences were observed between AD and sham rats. Chen et al. (2019) study showed similar finding to our analysis.

The animal behavioral test is essential to understand the bases of neurologic and psychological disorders (Bello-Arroyo et al., 2018). The Y-maze test was implemented to assess immediate spatial working memory of animals (Xu et al., 2018). Because of the simple structure and convenient operation of automated Y-maze applications, more and more animal experiments have adopted the Y-maze to explore the learning and memory of animals (Ru and Liu, 2018). The numbers of arm entries and time spent in the new arm have been identified as well indices of short-term spatial memory (Lewis et al., 2017). The spontaneous alternation behavior, the time spent in the new arm, total distance and the total new arm distance were measured to assess the learning ability of the rats (Conrad et al., 2003).

Crescenzi et al. (2017) revealed that a reduced glutamate chemical exchange saturation transfer (GluCEST) in 3.0 ppm occurred in the subhippocampal fields of AD. Our previous study had found the best parameters for scanning APT (Shen et al., 2017). So, in this study, the APT signals most of the contribution comes from the mobile protein with amide proton, rather than glutamate and glutamine. The increased GFAP in pathology and the loss of neurons in HE staining indicated that amyloid protein toxicity led to glial cell proliferation and neuronal loss (Trias et al., 2017), indicating that the modeling was successful and abnormal protein deposition was the cause of APT signal change.

### Limitations

The calculation of APT CEST metric in this study was conventional MTRasym. More specific analysis using the fitting algorithm, which we are currently still working on, would reduce the impact of several unwanted contributors of the APT contrast and make the result more accurate.

## Conclusion

This report demonstrated the value of APT_*SAFARI*_ as a non-invasive MRI technique for assessment of AD rat model. The non-invasive nature of APT data collection may allow for a relatively easy translation into the clinical setting. However, further standardization and improvement are required to provide useful diagnostic data within clinically feasible imaging times. We demonstrated abnormality in AD models compared to the sham operated controls, as confirmed by subsequent analysis of histological examinations. In summary, this is the first report of using APT technique with SAFARI method to detect AD. Our study provides evidence of the feasibility of APT imaging in the detection of cerebral abnormality in AD model and it has great potential for clinical application.

## Data Availability

All datasets generated for this study are included in the manuscript or the supplementary files.

## Ethics Statement

All animal experiments were performed according to the guidelines of the National Institutes of Health guide and approved by the Ethics Committee of Shantou University Medical College.

## Author Contributions

RRW and PC were responsible for the study design, acquisition and drafting the manuscript. ZS and ZD was responsible for interpretation of data. BZ undertook the immunohistochemistry analyses. GX and GL performed the CEST data analysis and CEST imaging processing. RRW, YT, and YL built the AD model. YC, XZ, and LL assisted in Y-maze test. RHW was responsible for the study concept and design, study supervision, obtaining funding.

## Conflict of Interest Statement

The authors declare that the research was conducted in the absence of any commercial or financial relationships that could be construed as a potential conflict of interest.
